# Single Abrikosov vortices as quantized information bits

**DOI:** 10.1038/ncomms9628

**Published:** 2015-10-12

**Authors:** T. Golod, A. Iovan, V. M. Krasnov

**Affiliations:** 1 Department of Physics, Stockholm University, AlbaNova University Center, SE-10691 Stockholm, Sweden

## Abstract

Superconducting digital devices can be advantageously used in future supercomputers because they can greatly reduce the dissipation power and increase the speed of operation. Non-volatile quantized states are ideal for the realization of classical Boolean logics. A quantized Abrikosov vortex represents the most compact magnetic object in superconductors, which can be utilized for creation of high-density digital cryoelectronics. In this work we provide a proof of concept for Abrikosov-vortex-based random access memory cell, in which a single vortex is used as an information bit. We demonstrate high-endurance write operation and two different ways of read-out using a spin valve or a Josephson junction. These memory cells are characterized by an infinite magnetoresistance between 0 and 1 states, a short access time, a scalability to nm sizes and an extremely low write energy. Non-volatility and perfect reproducibility are inherent for such a device due to the quantized nature of the vortex.

## Abstract (web summary)

Superconductors can be used in future supercomputers because they can greatly reduce power consumption and facilitate nonvolatile quantized states that are ideal for Boolean logics. Here, the authors provide a proof-of-concept RAM memory based on a single Abrikosov vortex as bit.

## Graphical abstract

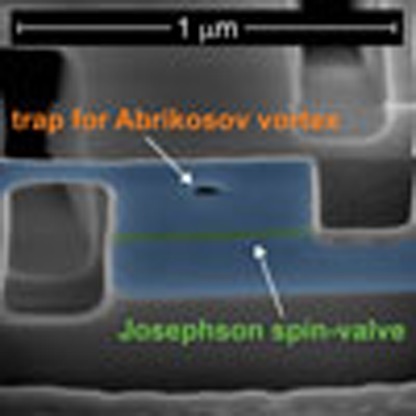

Growing power consumption in supercomputers is becoming an increasing problem. For an exaflop computer it is predicted to be on a 100-MW level^1^. Management of such a power would be very difficult for the present semiconductor-based technology. The corresponding maintenance costs exceed the cost of refrigeration to cryogenic temperatures. Therefore, efforts are taken for the development of superconducting computers^2^, which would not only drastically decrease the consumed power but could also greatly increase the operation speed^3^,^4^,^5^. Lack of suitable cryogenic random access memory (RAM) is the ‘main obstacle to the realization of high-performance computer systems and signal processors based on superconducting electronics'^6^.

Requirements for a cryogenic RAM are rather demanding^2^,^6^. Owing to a limited cooling power at low temperatures, it should be non-volatile with zero static consumption and extremely low access energy ≲10^−18^ J bit^−1^ and, of course, it should be fast and should have high density. Today the most developed superconducting RAM is based on a storage of a flux quantum Φ_0_ in a SQUID (superconducting quantum interference device) loop^7^. However, such a RAM has a major scalability problem^6^. Recently, several proposals have been made aiming to employ hybrid superconductor/ferromagnet (S/F) structures in RAM^8^,^9^,^10^,^11^,^12^. In this case the information is stored in magnetic layers, as in magnetic random access memory (MRAM)^13^. Read/write operation and limits of scalability for such a hybrid RAM remain to be studied.

Quantized Abrikosov vortex (AV) represents the smallest magnetic bit Φ_0_ in a superconductor with a characteristic size given by London penetration depth *λ*∼100 nm. The AV can be easily manipulated by short current pulses and can be detected in different ways^14^,^15^,^16^,^17^. Therefore, it is interesting to investigate a possibility of using a single AV as a memory bit^18^,^19^.

In this work we provide a proof of concept for Abrikosov vortex-based random access memory (AVRAM) memory cell, in which a single vortex is used as an information bit. We demonstrate high-endurance write operation and two different ways of read-out using a spin valve or a Josephson junction. AVRAM cells are characterized by an infinite magnetoresistance (MR) between 0 and 1 states, a short access time, a scalability to nm sizes and an extremely low write energy. Non-volatility and perfect reproducibility are inherent for such a device due to the quantized nature of the vortex.

## Results

### AVRAM with a Josephson spin-valve read-out

Here we demonstrate two prototypes of AVRAM cells with different geometries and read-out principles: Nanoscale Josephson spin valves (JSVs)^20^,^21^ and planar Josephson junctions^17^,^22^,^23^ with a vortex trap. Details of fabrication, parameters of the studied devices and experimental set-up can be found in Methods.

**Figure 1:**
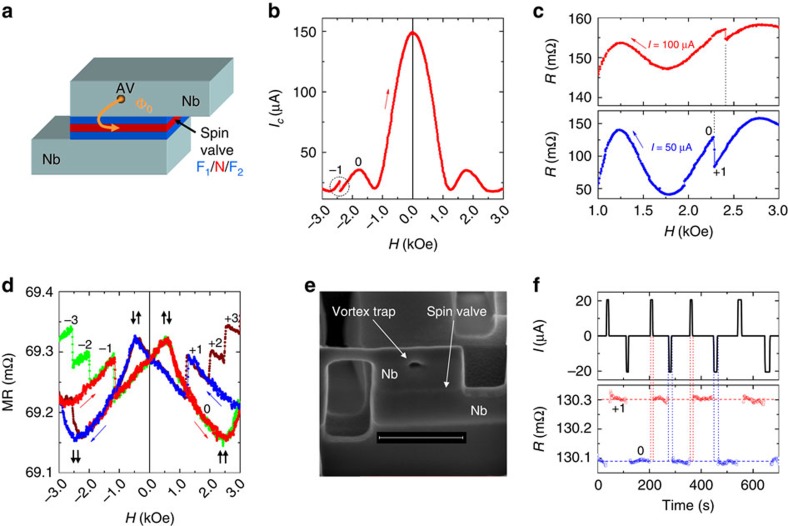
Operation of a memory cell with a Josephson spin-valve read-out. (**a**) Sketch of a Josephson spin-valve device. (**b**) Magnetic field dependence of Josephson critical current through the JSV#1 at *T*=2.4 K. A jump, marked by a circle, is due to antivortex exit. (**c**) Magnetic field dependence of a.c. resistance for JSV#1 at *I*_a.c._=50 (bottom plot) and 100 (top plot) μA. It is seen that the vortex exit field depends on the current. (**d**) High-bias spin-valve MR for a JSV#2 at *T*=1.8 K. Multiple branches at high fields are caused by entrance of vortices. The number of vortices (0–3) is marked at each branch. (**e**) Scanning electron microscopy image of JSV#3 AVRAM cell with a vortex trap. Scale bar, 500 nm. (**f**) Demonstration of write and erase operations for the same cell at *T*=1.8 K and *H*=2.4 kOe. Top plot shows applied current pulses, bottom plot the a.c. resistance.

Figure 1a shows a sketch of a nanoscale JSV, in which junction barrier consists of a spin valve. Such a device allows simultaneous analysis of both the Josephson current *I*_c_ and the spin-valve MR^20^, which facilitate two ways of AV detection (Supplementary Fig. 1 and Supplementary Note 1). Figure 1b shows *I*_c_(*H*) modulation for a JSV#1. Field, parallel to the junction plane, is swept from a sufficiently large negative value, at which one antivortex is trapped in a Nb electrode of the JSV. An abrupt jump in *I*_c_, marked by a circle, occurs on exit of the antivortex^17^. Figure 1c shows resistances *R*(*H*) for the same JSV measured with different a.c. currents *I*_a.c._≳*I*_c_ for the field sweep from positive to negative. *R*(*H*) reflects Fraunhofer modulation of *I*_c_(*H*). The resistance jumps on exit of the vortex. The field at which AV exits depends on applied current^15^,^17^.

Figure 1d shows spin-valve MR for the JSV#2 measured at large current *I*_a.c._>>*I*_c_. Soft hysteresis around *H*=0 is due to rotation of magnetization of the two F layers from parallel to antiparallel states^20^, as marked by thick vertical arrows. At higher fields |*H*|>1 kOe another type of hysteresis with abrupt switching between discrete states appears. It is due to one-by-one entrance/exit of AVs into Nb electrodes^15^. Stray fields from AV offset the spin valve by Δ*H*∼Φ_0_/*d*_SV_*w*, where *w* is the width and *d*_SV_ is the total thickness of the spin valve. For our nanoscale JSV (*w*=200 nm and *d*_SV_=30 nm) the offset Δ*H*∼1 kOe is remarkably large and easily measurable.

For a better control of the AV position we introduced an artificial vortex trap in Nb, as shown in Fig. 1e. This greatly improved the stability of write operation. Figure 1f demonstrates the controllable write and erase operation for this memory cell. The top plot shows a current train consisting of positive and negative pulses. The bottom plot shows the corresponding JSV resistance. The high/low resistance corresponds to states with/without AV. It is seen that the AV is introduced by positive and removed by negative pulses of small amplitudes ∼20 μA.

### AVRAM with a planar Josephson junction read-out

**Figure 2:**
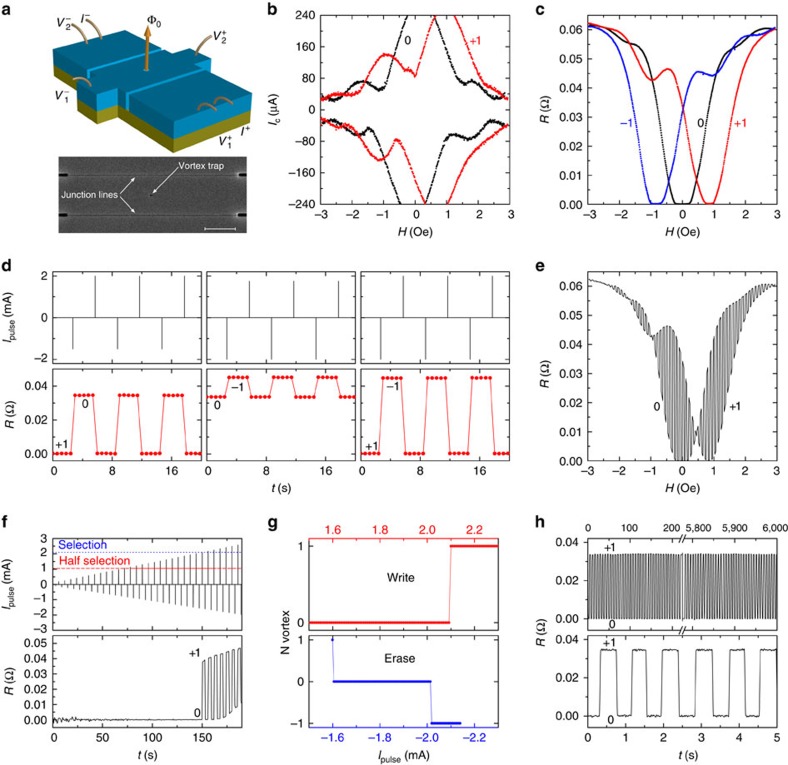
Operation of a memory cell with planar Josephson junctions. (**a**) Scanning electron microscopy (SEM) image and sketch of a planar AVRAM cell with a vortex trap and two read-out planar Josephson junctions. The scale bar in SEM image is 1 μm. (**b**) Magnetic field modulation of the critical current for the first read-out junction without (black) and with a vortex (red). (**c**) Magnetic field dependence of a.c. resistance without a vortex (black), with a vortex (red) and with an antivortex (blue line). (**d**) Demonstration of write and erase operations by current pulses of different amplitudes. (**e**) Demonstration of controllable 0–1 switching in a broad field range. (**f**) Evolution of the device state on applying a pulse train with growing amplitude. Note excellent half-selection stability. (**g**) Dependence of the final state on the pulse amplitude. (**h**) Demonstration of high-endurance 0–1 switching at zero applied field.

In Fig. 2 we analyse operation of another type of a memory cell based on a particularly simple planar structure. Figure 2a shows a scanning electron microscopy image and a sketch of the cell. It consists of a cross-shaped superconducting film with a vortex trap and two read-out planar Josephson junctions^22^. AV generates a 0−*π* phase shift in the junctions, which strongly affects the *I*_c_(*H*) pattern^17^. This is demonstrated in Fig. 2b for one of the junctions. The second junctions shows similar behaviour (Supplementary Fig. 2 and Supplementary Note 2) confirming that the signal originates from AV in the trap and not elsewhere. Figure 2c represents *R*(*H*) for different field sweeps. Three distinct states are seen: 0 vortex free (black); +1 with a vortex (red); and −1 with an antivortex (blue curve). The planar structure has two important advantages: first, 0 and ±1 states are stable at *H*=0; and second, AV is introduced at a very low field ∼1 Oe due to a large demagnetization factor (flux-focusing effect)^23^ (field is applied perpendicular to the plain).

Figure 2d demonstrates write and erase operation with short pulses of different amplitudes. Measurements are done at *H*∼0.8 Oe to distinguish +1 and −1 states. It is seen that depending on the pulse height and direction we can either switch between 0 and +1 (left plot), 0 and −1 (middle plot), or −1 and +1 (right plot) states. Application of additional pulses in the same direction does not change the state of the device.

Figure 2e represents *R*(*H*) measured on simultaneous sweeping of magnetic field and applying current pulses. It is seen that a controllable switching between 0 and +1 states occurs in the whole field range, implying that external magnetic field is not needed and does not hinder operation of such AVRAM cell. Moreover, *H*=0 is one of the optimal points for having the largest MR.

Figure 2f demonstrates switching of the device, starting from the 0 state, for a pulse train with increasing current amplitude at *H*=0. It is seen that the vortex is introduced above a selection current *I*≃2.1 mA. RAM should be stable with respect to half-selection current, which is indicated by the dashed line in the top plot. It is seen that not only half-selection but also 12 other pulses between half-selection and selection do not cause switching of the memory state. Figure 2g demonstrates dependence of the final state on the pulse amplitude. The top plot corresponds to write operation starting from the 0 state. The bottom plot shows erase operation starting from the +1 state. At *I*<−1.6 mA the device switches to 0 state and at *I*<−2.0 mA, to −1 state. Abruptness of the threshold currents and absence of sub-threshold switching indicate excellent half-selection stability of both write and erase operations.

In Fig. 2h we analyse 0–1 write–erase operation at zero field. Top plot demonstrates high-endurance operation for almost 2 h (Supplementary Fig. 3 and Supplementary Note 3). Bottom plot shows a time sequence for the shortest (instrumentation limited) pulse duration of ∼17 μs. Both states are stable and non-volatile. Note that because *R*=0 in the 0 state, the device is characterized by infinite MR^24^. The remarkable reproducibility of switching is the consequence of quantized nature of the vortex.

## Discussion

Finally we would like to discuss perspectives and limitations of the AVRAM.

Scalability. The main problem of existing memory based on SQUID^7^ is the lack of scalability^6^: the required current *I*∼Φ_0_/*L* increases with decreasing the loop inductance *L* on miniaturization of the loop. In AVRAM the applied current density *J* should exceed the depinning current density *J*_p_, which is a material constant. Therefore, threshold current will scale down with the cross-section of the device. Consequently, the AVRAM is scalable at least down to the vortex size *λ*∼100 nm. We demonstrated this on our JSV type nanostructures (Fig. 1). Vortex states exist even in smaller structures down to the coherence length *ξ*∼10 nm (ref. 25), but stabilization of the vortex state in so small structures would require significant magnetic fields, as already seen for our nanoscale JSV.

Speed. AV is moved by Lorentz force F_L_=Φ_0_*J*/*c* (per unit length of the vortex). For a film of width *w*>>*λ* the time required to move the vortex in, or out is *t*=*w*/2*v*, where *v*=Φ_0_*J*/*cη* is the drift velocity of the vortex and *η*=Φ_0_*H*_*c*2_/*c*^2^*ρ*_n_ is the viscosity of the vortex motion^26^. Thus, *t*=*H*_c2_*w*/2*cJρ*_n_. Taking the upper critical field *H*_c2_=20 kOe, *w*=1 μm, *J*_p_=10^6^ A cm^−2^ and normal resistivity *ρ*_n_=10^−5^ Ω cm, typical for sputtered Nb films^27^, we obtain *t*=1 ns. For mesoscopic structures *w*≲*λ*, entrance and exit of AV is not due to viscous motion, but due to switching between metastable states^25^,^28^,^29^. At the selection current a barrier between 0 and 1 states turns to zero and the switching time is limited by the relaxation time of the order parameter *τ*_r_ (ref. 30), which can be less than a nanosecond. Note that for planar junctions *w* should be compared with Pearl length *λ*_P_=Λ^2^/*d* so that even few-μm-wide planar junctions with *d*<*λ* may be in the mesoscopic state^23^. Ultimately the operation time is limited by propagation of a current pulse ∼100 ps due to kinetic inductances of electrodes^6^.

Write/erase energy. Cryogenic RAM must have very low access energy. For the AV the minimum write/erase energy is equal to the work done by Lorentz force to move the vortex across the structure: *E*=Φ_0_*I*/2*c*. Even for a fairly high-pulse current *I*=1 mA we get an acceptably low *E*=10^−18^ J.

Thus, we demonstrated that AV can be used as an information bit. The two studied devices utilize different detectable quantities of the vortex: the stray field and the phase shift. Our JSVs demonstrate the possibility of scaling to sub-micron sizes and having very small write currents. The apparent disadvantage, however, is the required significant magnetic field: the smaller the structure the larger the field. Here planar junctions provide a great advantage because they can operate at zero field. The field scale in planar structures is reduced by the flux-focusing effect due to large demagnetization factor. We emphasize that excellent reproducibility, half-selection stability and high endurance are inherent for AVRAM due to quantized nature of the vortex, which prevents intermediate states. Finally, we note that similar devices can be used for doing basic Boolean operations. For example, AND or OR operations can be performed by sending current pulses into serial or parallel connection of two AVRAM cells, respectively. The pulse amplitude should be such that it causes switching only if maximum one, but not both, of the cells are in the high-resistance state. This should be possible due to excellent selectivity of switching, shown in Fig. 2g. Therefore, we conclude that such devices have a large potential for application in digital cryoelectronics.

## Methods

### Sample fabrication

Thin film heterostructures were deposited by magnetron sputtering. Films were first patterned into μm-size bridges by photolithography and ion etching and subsequently nano-patterned by focused ion beam (FIB). Nanoscale JSV devices are made by three-dimensional nanosculpturing with FIB^17^,^20^,^21^. Planar Nb/CuNi/Nb junctions were made by cutting Cu_0.47_Ni_0.53_/Nb (50/70 nm) double layers by FIB^17^,^22^,^23^. Vortex traps for both types of memory cells (a hole with a diameter 30–50 nm) were also made by FIB.

### Josephson spin-valve parameters

We studied several JSV devices with dissimilar F layers made of diluted (CuNi) or strong (Py, Co and Ni) ferromagnets: JSV#1 Nb/Cu/Py/Cu/Co/Cu/Nb (200/5/2/10/2/5/200 nm) with sizes 200 nm × 1.8 μm, JSV#2 Nb/Cu_0.5_Ni_0.5_/Cu/Cu_0.4_Ni_0.6_/Nb (200/10/10/10/200 nm) with sizes 200 nm × 1 μm and JSV#3 Nb/Ni/Cu/Cu_0.4_Ni_0.6_/Nb (200/7/10/10/200 nm) with sizes 135 × 530 nm^2^. All of them were showing similar behaviour.

### Experimental

Measurements were performed in closed-cycle cryostats. Critical currents were determined from current–voltage characteristics by finding the extreme currents within a voltage range of a certain threshold level. For measurements of resistance, a set of synchronized lock-in amplifiers built around a field-programmable gate array was used.

## Additional information

**How to cite this article:** Golod, T. *et al.* Single Abrikosov vortices as quantized information bits. *Nat. Commun.* 6:8628 doi: 10.1038/ncomms9628 (2015).

## Supplementary Material

Supplementary Information Supplementary Figures 1-3 and Supplementary Notes 1-3
