# Supplementary material for: Single Abrikosov vortices as quantized information bits
Source: Nat Commun. 2015 Oct 12;6:8628. doi: 10.1038/ncomms9628 (PMC4633956; doi:10.1038/ncomms9628)
Supplement: Supplementary Information — Supplementary Figures 1-3 and Supplementary Notes 1-3 [file ncomms9628-s1.pdf]

Supplementary information:

SUPPLEMENTARY FIGURES

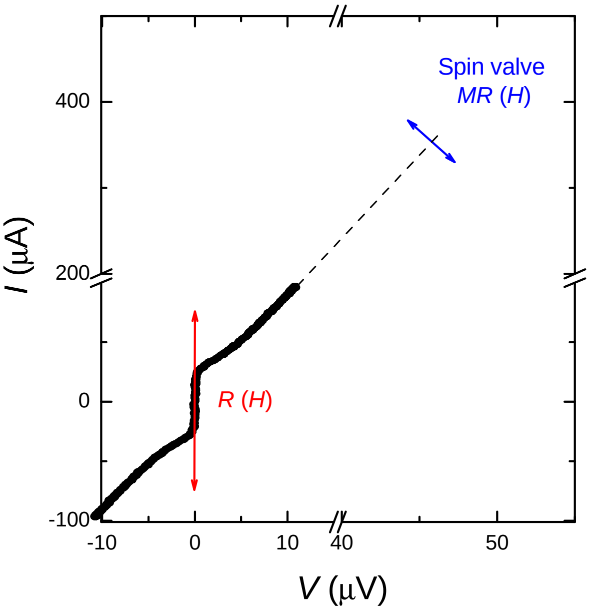

Supplementary Figure 1. **Clarification of two ways of probing local magnetic fields by a Josephson spin valve.** Typical current-voltage characteristics of a JSV. Arrows indicate two ways of probing local magnetic field by resistive measurements. Low bias resistance $R(H)$, measured with $I_{\mathrm{ac}} \gtrsim I_{\mathrm{c}}$ in a strongly non-linear part of the I-V, reflects Fraunhofer modulation of $I_{\mathrm{c}}(H)$ due to Josephson effect. High bias resistance $I_{\mathrm{ac}} \gg I_{\mathrm{c}}$, measured in the linear part of the I-V, represents the spin-valve magnetoresistance that depends on relative orientation of two ferromagnetic layers.

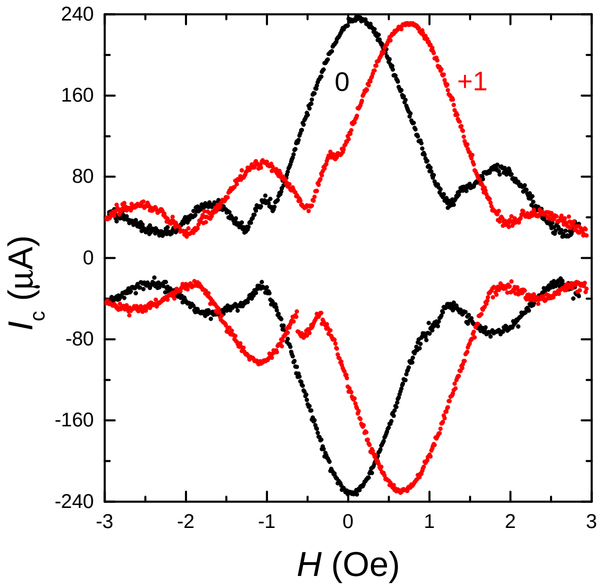

Supplementary Figure 2. **Magnetic field modulation of the critical current for the second readout junction.** Black curve represents vortex-free 0 state and red curve +1 state with one vortex in the trap. The behavior is similar to that for the first junction, shown in Fig. 2b.

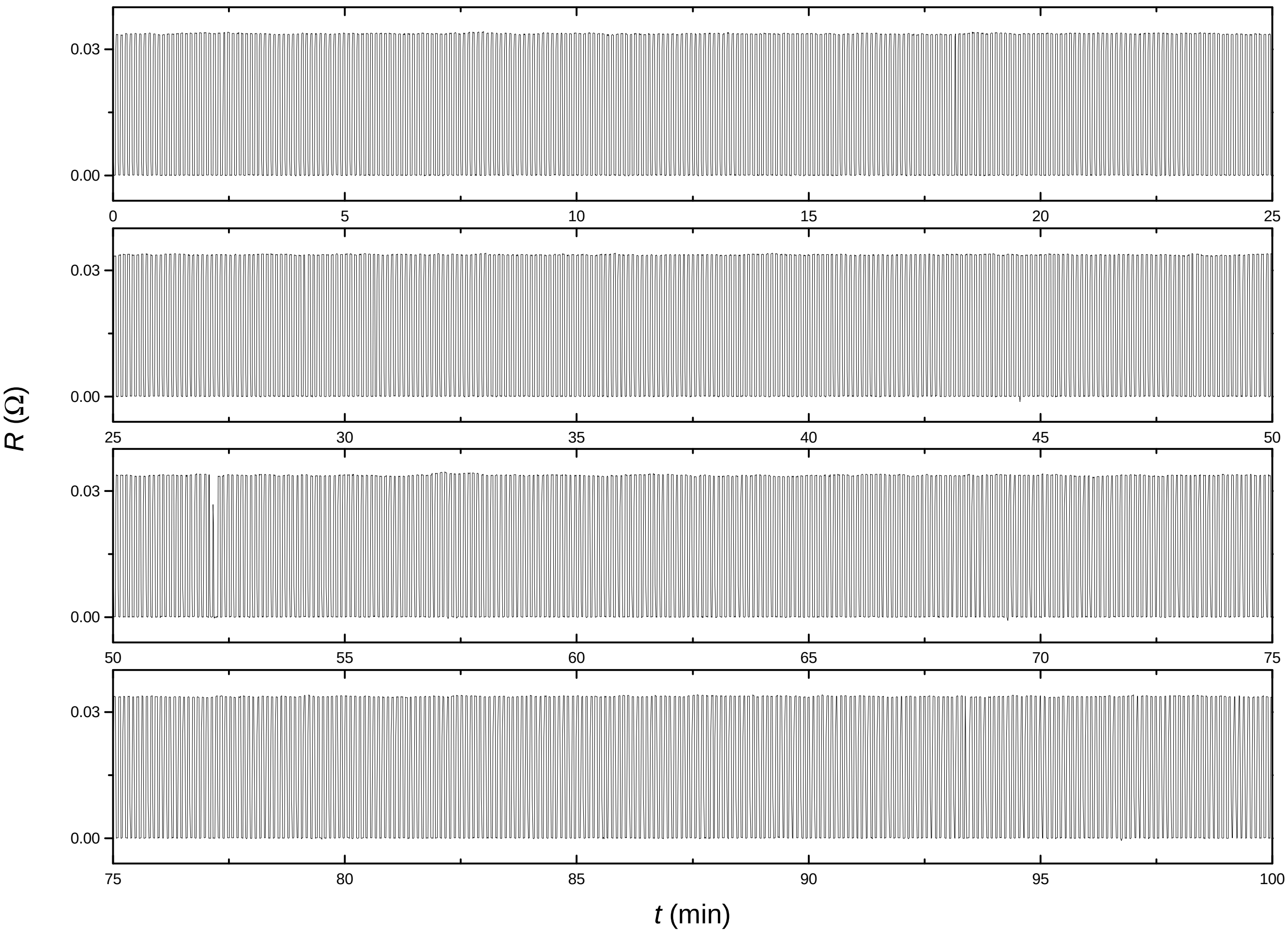

Supplementary Figure 3. **Demonstration of high endurance operation at zero magnetic field.** 100 minute fault-free 0-1 switching of a planar AVRAM cell. Note infinite magnetoresistance of the device: $[R(1) - R(0)]/R(0) = \infty$.

SUPPLEMENTARY NOTES

Supplementary Note 1. Josephson spin-valve operation

Supplementary Figure 1 shows the I-V characteristics of Nb/CuNi/Cu/CuNi/Nb JSV at $T = 1.8$ K and $H = 0$. It has a clear critical current I_c followed by an Ohmic branch, representing the resistance of the spin valve. The JSV exhibits two types of responses to magnetic field.

First, the Josephson critical current oscillates as a function of magnetic field due to flux quantization in the junction. As in conventional Josephson junctions, the $I_c(H)$ follows Fraunhofer pattern with zeroes at integer number of Φ_0 in the JSV (see Fig. 1b). Resistance R measured in the non-linear part of the I-V with the small ac-current $I_{ac} \gtrsim I_c$, as marked by red arrows in Supplementary Figure 1, depends on the magnitude of $I_c(H)$ and, therefore, carries information about the total flux Φ/Φ_0 in the device, as seen from Fig. 1c.

Second, the spin valve resistance depends on a relative orientation of magnetization of the two ferromagnetic layers with minima in the parallel state and maxima in the anti-parallel state, as shown in Fig. 1d. The orientation depends on magnetic field. Therefore, the spin valve MR is sensitive to applied magnetic field. It can be measured by applying large current $I_{ac} \gg I_c$, as shown by the blue arrows in Supplementary Figure 1. The corresponding data is shown in Fig. 1d.

This provides two different ways of probing the local magnetic field from resistance measurements, which can be used for readout of AVRAM. More details about operation of our JSV can be found in Ref. [16].

Supplementary Note 2. Characteristics of planar junctions

Due to cross-like structure of the planar cell, we could simultaneously and independently measure both readout junctions. In this case the current is sent sequentially through both junctions. Two additional electrodes between the junctions are used for independent measurement of voltages across both junctions.

Supplementary Figure 2 represents $I_c(H)$ modulation for the second readout junction at the same planar device. It was obtained simultaneously with the data shown in Fig. 2b under the same conditions. The second readout junction is not needed for operation of the AVRAM cell. In the test device it was used solely to investigate the origin of the detected signal. Similar behavior of both readout junctions indicates that the signal originates from the vortex in the trap, and not elsewhere.

Supplementary Note 3. Demonstration of high endurance operation

We observed fault-free write/erase operation of the planar AVRAM cell at $H = 0$ over a period of several hours. An example is shown in Supplementary Figure 3.
